# Histone code and long non-coding RNAs (lncRNAs) aberrations in lung cancer: implications in the therapy response

**DOI:** 10.1186/s13148-017-0398-3

**Published:** 2017-09-08

**Authors:** Abril Marcela Herrera-Solorio, Leonel Armas-López, Oscar Arrieta, Joaquín Zúñiga, Patricia Piña-Sánchez, Federico Ávila-Moreno

**Affiliations:** 1 Cancer Epigenomics and Lung Diseases Laboratory-12 (UNAM-INER), Biomedicine Research Unit (UBIMED), Facultad de Estudios Superiores (FES)-Iztacala, Universidad Nacional Autónoma de México (UNAM), Mexico State, Mexico; 2 Thoracic Oncology Unit and Laboratory of Personalized Medicine, Instituto Nacional de Cancerología (INCan), Mexico City, Mexico; 3 Research Unit, Instituto Nacional de Enfermedades Respiratorias (INER), Ismael Cosío Villegas, Mexico City, Mexico; 4 Molecular Oncology Laboratory, Unidad de Investigación Médica en Enfermedades Oncológicas (UIMEO), CMN., SXXI., IMSS, Mexico City, Mexico

## Abstract

Respiratory diseases hold several genome, epigenome, and transcriptional aberrations as a cause of the accumulated damage promoted by, among others, environmental risk factors. Such aberrations can also come about as an adaptive response when faced with therapeutic oncological drugs. In epigenetic terms, aberrations in DNA methylation patterns, histone code marks balance, and/or chromatin-remodeling complexes recruitment, among Polycomb Repressive Complex-2 (PRC2) versus Trithorax (TRX) Activator Complex, have been proposed to be affected by several previously characterized functional long non-coding RNAs (lncRNAs). Such molecules are involved in modulating and/or controlling lung cancer epigenome and genome expression, as well as in malignancy and clinical progression in lung cancer.

Several recent reports have described diverse epigenetic modifications in lung cancer cells and solid tumors, among others genomic DNA methylation and post-translational modifications (PTMs) on histone tails, as well as lncRNAs patterns and levels of expression. However, few systematic approaches have attempted to demonstrate a biological function and clinical association, aiming to improve therapeutic decisions in basic research and lung clinical oncology. A widely used example is the lncRNA HOTAIR and its functional histone mark H3K27me3, which is directly associated to the PRC2; however, few systematic pieces of solid evidence have been experimentally performed, conducted and/or validated to predict lung oncological therapeutic efficacy.

Recent evidence suggests that chromatin-remodeling complexes accompanied by lncRNAs profiles are involved in several comprehensive lung carcinoma clinical parameters, including histopathology progression, prognosis, and/or responsiveness to unique or combined oncological therapies. The present manuscript offers a systematic revision of the current knowledge about the major epigenetic aberrations represented by changes in histone PTMs and lncRNAs expression levels and patterns in human lung carcinomas in cancer drug-based treatments, as an important comprehensive knowledge focusing on better oncological therapies. In addition, a new future direction must be refocusing on several gene target therapies, mainly on pharmaceutical EGFR-TKIs compounds, widely applied in lung cancer, currently the leading cause of death by malignant diseases.

## Background

1.6 million deaths occur by lung malignant diseases each year, remaining as the leading cause of death by oncological diseases worldwide [1]. Lung cancer has traditionally been classified in different histopathological groups such as small cell lung carcinomas (SCLC) and non-small cell lung carcinomas (NSCLC) with an average of 10–15% and 85–90% of total cases, respectively [2]. NSCLC has been sub-classified into specific clinical and histopathological subtypes including adenocarcinomas (AD), squamous cell lung carcinomas (SCC), and large cell lung carcinomas (LCC) [2, 3].

Lung cancer has been strongly associated with tobacco smoking averaging 90% of total cases; However epidemiologically associated to SCC histology type; whereas AD has been associated with lower tobacco smoking exposure rate [2, 4]. Other occupational carcinogenic exposure includes asbestos, arsenic, vinyl chloride, nickel chromates, coal products, mustard gas, and chlorine methyl-ethers which could be associated with 9–15% of total cases. In recent years, it has been estimated that air pollution is contributing to 1–2% of lung cancer total cases [4].

Previous reports support the effect of genetic, transcriptional, and epigenetic aberrations through lung cancer induction, initiation, promotion, and progression. These molecular alterations are mainly involved in homeostasis disruption, focusing on genetic expression “transcription” of cellular key genes, including oncogenes, tumor suppressor genes, as well as DNA damage repair, replication, and cellular apoptosis mechanisms [5, 6]. These can be partially explained by epigenetic factors, such as transitory and/or permanent changes on DNA methylation patterns, histone code modifications due to acetylation, methylation, phosphorylation, ubiquitination, etc. [7]. Alterations for chromatin remodeling mechanisms, some of them associated or functionally involved with long non-coding RNAs “lncRNAs” [8], are key factors that affect histone code changes or permanently contribute to the generation of histone code aberrations in lung cancer.

### Histone code aberrations in lung cancer

Recent evidence reveals that different environmental risk factors, including diet, stress, physical activity, aside from alcohol consumption, smoking, air pollution, and environmental heavy metals (nickel, cadmium, arsenic, etc.,), are increasing cellular production of reactive oxygen species “ROS” [9]. These highly reactive oxygen species definitively affect DNA methylation status, histone code changes, and chromatin remodelation mechanisms coupled or non-coupled to lncRNAs aberrant patterns [10]. Additionally, they are involved in post-translational modifications (PTMs) on histone tails, which have been characterized with high basic amino acids content, providing a strong negative charge throughout the genome and in specific regulatory DNA domains (e.g. Sequence Promoters). Each histone consists of one terminal carboxyl (COOH) domain, which carries out histone-histone and histone-DNA interactions, and one amino (NH_2_) terminal domain, carrying out lysine residues covering nucleosome structures [11]. NH_2_ terminal domains are sensitive to proteases and provides a surface that could interact with the modifying enzymes, assessing PTMs, and controlling RNA polymerase II (RNA Pol II) and transcription factors accessibility at DNA sequence domains [12, 13].

Nowadays, several PTMs, such as phosphorylation of serines (S) and threonines (T), methylation of arginines (R), as well as acetylation, methylation, ubiquitination, sumoylation, and ADP-ribosylation of lysines (K) [14, 15], and recently lysines crotonylation [16], have been identified as key histone tail modifications. They are frequently involved in chromatin relaxation versus condensation, in a reversible dynamic mechanism altering genetic and genome-wide expression profiles [15]. All of these biochemical changes have been involved in two major epigenetic events: (i) altering the electrostatic charge of the histones and its binding properties to genomic DNA control domains, affecting the transcriptional machinery assembly, and (ii) creating a new common protein surfaces which in turn recruits additional transcriptional complexes through genome or specific DNA regulatory domains, similar to what occurs by spread heterochromatin protein 1 (HP1), accompanied by a specific PTM profile [17, 18].

PTMs have been epigenetically classified based on their functional activation versus repression status. The activation state has predominantly been based on H3 acetylation on lysine 9 (H3K9ac), either di- or tri-methylation on lysines 4, 36, and 79 (H3K4me, H3K36me, H3K79me), as well arginine 17 methylation (H3R17me). Meanwhile, the repression status is also associated with transcriptional repression based on deacetylation and methylation processes at both, lysines 9 and 27 of histone H3 (H3K9me2/3 and H3K27me3), in addition to the H4K20 methylation process [15, 19, 20]. Concerning histone modifiers, enzymes such as acetyl-transferases (HATs), histone deacetylases (HDACs), sirtuins, histone methyl-transferases (HMTs), histone demethylases (HDMTs), histone kinases and phosphatases, ubiquitin ligases, and deubiquitinases, have been widely functionally characterized [15]. In this regard, a dynamic cooperation mechanism between DNA methylation and histone tails biochemical modifications has been reported to functionally play a crucial role in the molecular conformation of stability and packaging chromatin structures, DNA replication structures, and DNA repairing mechanisms. It is also reported to be involved in genome expression patterns, both transcriptionally and epigenetically controlled [21].

Based on the type of molecular alterations and/or aberrations in the recruitment by affinity of the epigenetic-enzyme complexes in physiological, as well as in malignant disease states, they have been functionally associated and/or involved in cancer development and histopathological progression [22].

First of all, DNA hypermethylation on CpGs islands or genetic control physical domains in others at several tumor-suppressor genes, oncogenes, and DNA repairing genes, and secondly, previous hypermethylation patterns associated with aberrant PTMs, including a global deacetylation process on histones H3 and H4. In this context, a lack of the trimethylation process on H3K4me2/3, in contrast to an increased trimethylation level on H3K9me3 and H3K27me3, is commonly observed in human lung cancer [6].

Some epigenetic changes in lung cancer have been described, such as an increased acetylation status for histone H2A, with an increased H3 global trimethylation process that has been seen in both NSCLC and SCLC histopathological types. Likewise, in solid lung tumors from patients with NSCLC, an excessive acetylation level on both histone marks H4K5 and H4K8, as well as hypoacetylation on H4K12/H4K16, accompanied with H4K20me3, in both lung malignant precursor lesions and particularly SCC lesions, has been identified [6].

Recent reports demonstrate that the combination of acetylated histone marks H2AK5ac/H3K9ac and H3K4me2 has a significant prognostic value in NSCLC [21]. Broeck and collaborators have shown aberrant PTMs patterns based on hyperacetylation for H4K5 and H4K8, as well as hypoacetylation on H4K12 and H4K16 in both AD and SCC histological types derived from NSCLC patients. On the other hand, the H4K20me3 mark, accompanied by a lower expression of the trimethyl-transferase Suv4-20h1/2 (also named KMT5B/C), was previously detected in lung precursor lesions with a significant association to a poor survival rate [23].

Immuno-histochemical expression of the PTMs marks H3K18ac, H4K12ac, H4R3me2, H3K4me2, and H3K27me3 were analyzed in a cohort of 97 patients with lung cancer, identifying a positive correlation between lung tumor differentiation stage with a H3K18ac, H4R3me2, and H3K27me3 pattern. In this study, reduced levels of H3K18ac and H3K27me3 were associated with better survival and prognosis rates in SCC histological cases [24], whereas a higher expression of H3K27me3 mark in NSCLC correlated with a better prognosis and longer overall survival rates [25]. Besides, stage I lung-AD patients with lower H3K9ac levels exhibit better prognosis [26].

Song and colleagues in 2012 identified a global status of PTMs on both H3 and H4 for AD and SCC lung malignant histological types (of a total 408 NSCLC cases) identifying a weak nuclear staining for H3K9ac, H3K9me3, and H4K16ac, excepting H4K20me3, related with tumor recurrence and distant metastasis. In this study, in general terms histone acetylation patterns (mainly H3K9ac) correlated with a better prognosis, whereas histone methylation patterns and/or negative nuclear staining were associated with a poor prognosis [27].

In addition, it has been recently demonstrated that a decreased H3K27me3 and increased H3K4me3 histone profile at gene promoter sequences of the Mesenchyme Homeobox-2 (MEOX2), HDAC9, and TWIST1 coding genes, as well additional genetical aberrations under aberrant epigenetic mechanisms, are associated or probably involved in poor clinical outcomes and lower survival rates, as well as involved in cancer-drug resistance mechanisms in NSCLC patients. The aforementioned genes are located in Chr:7p21, whose cytogenetic region has been reported to have a significant increased copy number variation (CNV) [28]. It is likely, therefore, that histone code aberrations are in functional coordination with lncRNAs and chromatin remodeling complexes function in lung cancer.

It has also been shown that a multi-subunit chromatin “nucleosome”-remodeling complex named SWI/SNF that uses the energy of ATP hydrolysis integrated of a catalytic subunit, either of BRG1 (also known as SMARCA4) or BRM (also known as SMARCA2), along with a variety of associated proteins may be involved in to modulate the recruitment of the complex and its activity to modify chromatin structure. This in turn regulates gene and genome expression, cell lineage, and organismal development [29, 30]. Different research groups have reported mutations which generally confer loss-of-functions or inactivate SWI/SNF subunits in nearly 20% of human cancers including lung cancer [29]. For example, loss of BRG1 and BRM expression has been reported in human NSCLC cell lines [30] both NSCLC and SCLC solid tumors, and this loss has been associated with a poor patient survival, when compared to patients with BRG1/BRM-positive lung tumors. This observation, suggests that BRG1/BRM act as tumor suppressor proteins [31]. Another SWI/SNF chromatin-modeling complex-member associated whose dysregulated expression level in NSCLC has been reported for the named ARID1A (AT-rich interactive domain 1A) gene. In this regard, Zhang and colleagues found that ARID1A expression was decreased in NSCLC tissues and such deregulation correlated with nodal metastasis, tumor, node, metastasis (TNM) stage, as well as, poor differentiation in cancer cell lines, promoting proliferation, colony formation ability, and inhibited paclitaxel-induced apoptosis [32]. In addition, it has also been recently reported that loss of the expression of SWI/SNF complex members such as ARID1A, ARID1B, and BAF47, correlates with a dedifferentiation histopathological phenotype in NSCLCs [33]. Finally, it has been experimentally demonstrated that inactivation of SWI/SNF complex members SMARCA4 (BRG1) or ARID1A has adverse effects on malignancy in early-stage lung adenocarcinomas, while loss of the histone methyltransferase Setd2 and subsequent loss of H3K36me3 led to the accelerated malignant progression at both early- and late-stage lung adenocarcinoma tumors [34]. All the evidence relayed above supports that chromatin remodeling complexes, nucleosome-histones, and lncRNAs are co-functionally involved in lung cancer therapy resistance and/or lung malignancy.

### LncRNAs aberrations in patients with lung malignant diseases

For a long time, RNA was considered a mere intermediary between DNA and proteins; however, it has been found that these messenger RNAs represent just a small fraction of the cellular total RNA; the, relatively recently-discovered, non-coding RNAs (ncRNAs), which constitute a class of RNA sequences that are transcribed but not encoded for proteins, have been implicated in the cellular control of multiple regulatory mechanisms including genetic expression, genome imprinting, epigenetic regulation, cellular proliferation, apoptosis, differentiation, migration and/or invasion, mRNA splicing processes, as well as being involved in human embryo development and chronic-degenerative diseases.

**Fig. 1:**
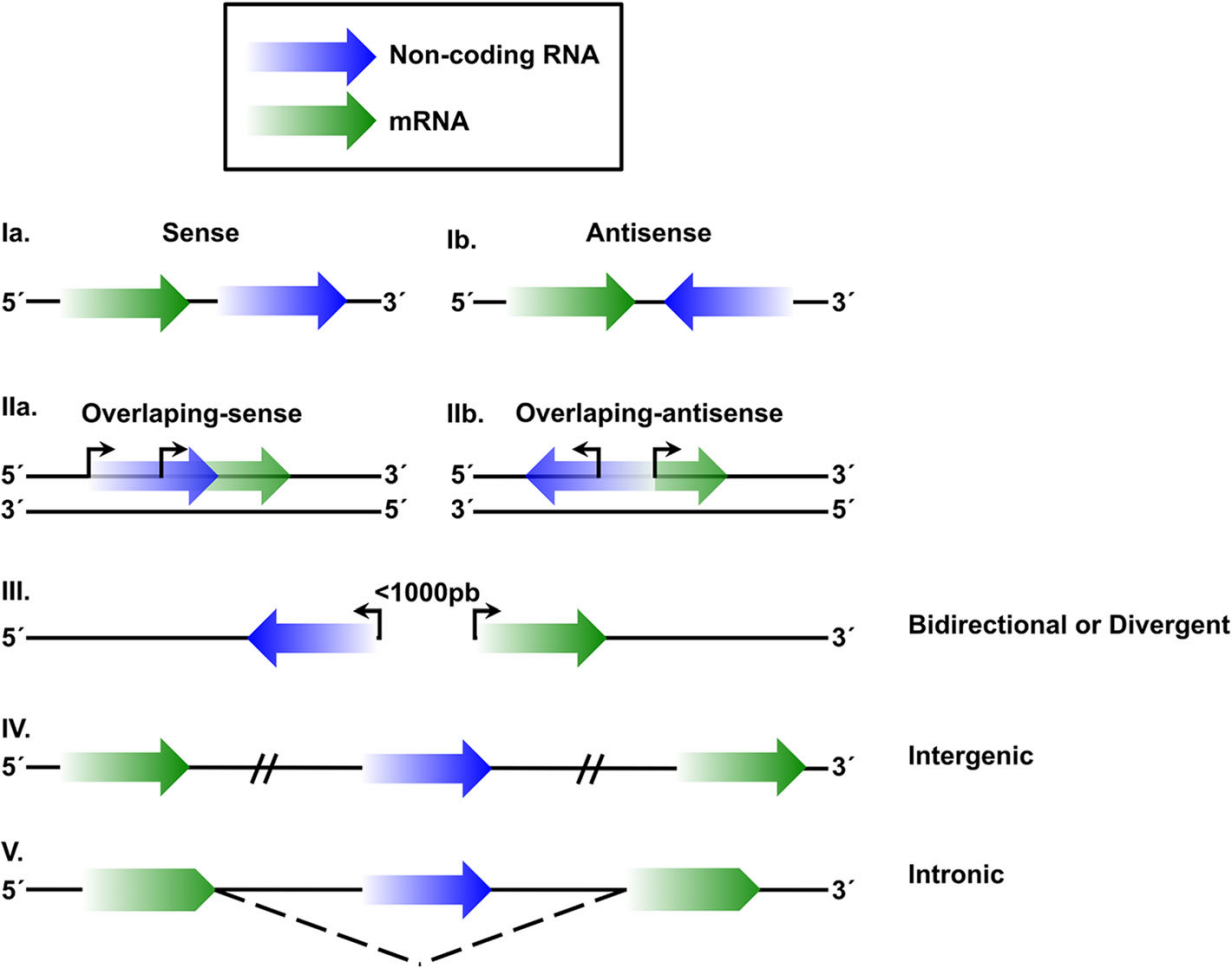
LncRNA classification based on their genomic location. Classification of lncRNAs (blue) based on their genomic position and in a relation to the neighboring genetic “mRNA” coding sequence (green). **Ia)** Sense lncRNAs. LncRNAs located in the same genetic chain with same and positive sense as the coding mRNA. **Ib)** Antisense lncRNAs. LncRNAs located on the opposite coding strand of the mRNA encoding sequences. **IIa)** Overlapping sense lncRNAs. Overlapped lncRNAs located in the same orientation sense with the mRNA encoding for protein. **IIb)** Overlapping antisense lncRNAs. Overlapped lncRNAs, located in opposite side with the mRNA encoding proteins. **III)** Bidirectional or divergent lncRNAs. LncRNAs located on the antisense strand (opposite to the neighboring lncRNA) that share or not promoter genetic sequences whose transcription start site (TSS) is close to the TSS of the coding mRNA sequences (< 1000 bp), meaning that they are transcribed in to the opposite direction with respect to the neighboring “mRNA” coding gene. **IV)** Intergenic lncRNA. LncRNAs which are transcribed from an intergenic region, transcribing from the sense or antisense strand. **V)** Intronic lncRNAs. LncRNAs located between the boundaries of the intronic and coding mRNA sequences. The arrow symbols meaning the transcription direction

In addition to the small (< 200 bp) ncRNAs which include micro-RNAs (miRNAs) and short interference RNAs (siRNAs), long ncRNAs (lncRNAs) have recently been studied and described in humans, with a wide range from > 200 bp and close to 100 kb [35, 36]. LncRNAs are transcribed by RNA polymerase II (RNA Pol II) in a genomic localization dependent manner, based on the nearest coding-transcript gene promoter, which may be classified as expressed transcripts in sense or antisense direction, as well as, focusing on uni- or bi-directional transcription sense related to its neighboring coding sequence genes, from intragenic, intronic, as well as, from intergenic regions (Fig. 1). LncRNAs are post-transcriptionally modified by 5′ capping and 3′ poly-adenylation processes [35–37]. In addition, meanwhile some lncRNAs are transcriptional and epigenetically regulated through hypomethylated DNA level, and histone marks H3K4me2/3, H3K9ac, H3K27me3 and H3K27ac, respectively. At the same time, they are closely associated with the transcription elongation process conducted by the enriched histone mark H3K36me3 throughout lncRNAs transcript bodies, suggesting in general molecular conditions where lncRNAs expression is epigenetically regulated as it conventionally occurs for the mRNAs coding genes [35].

**Fig. 2:**
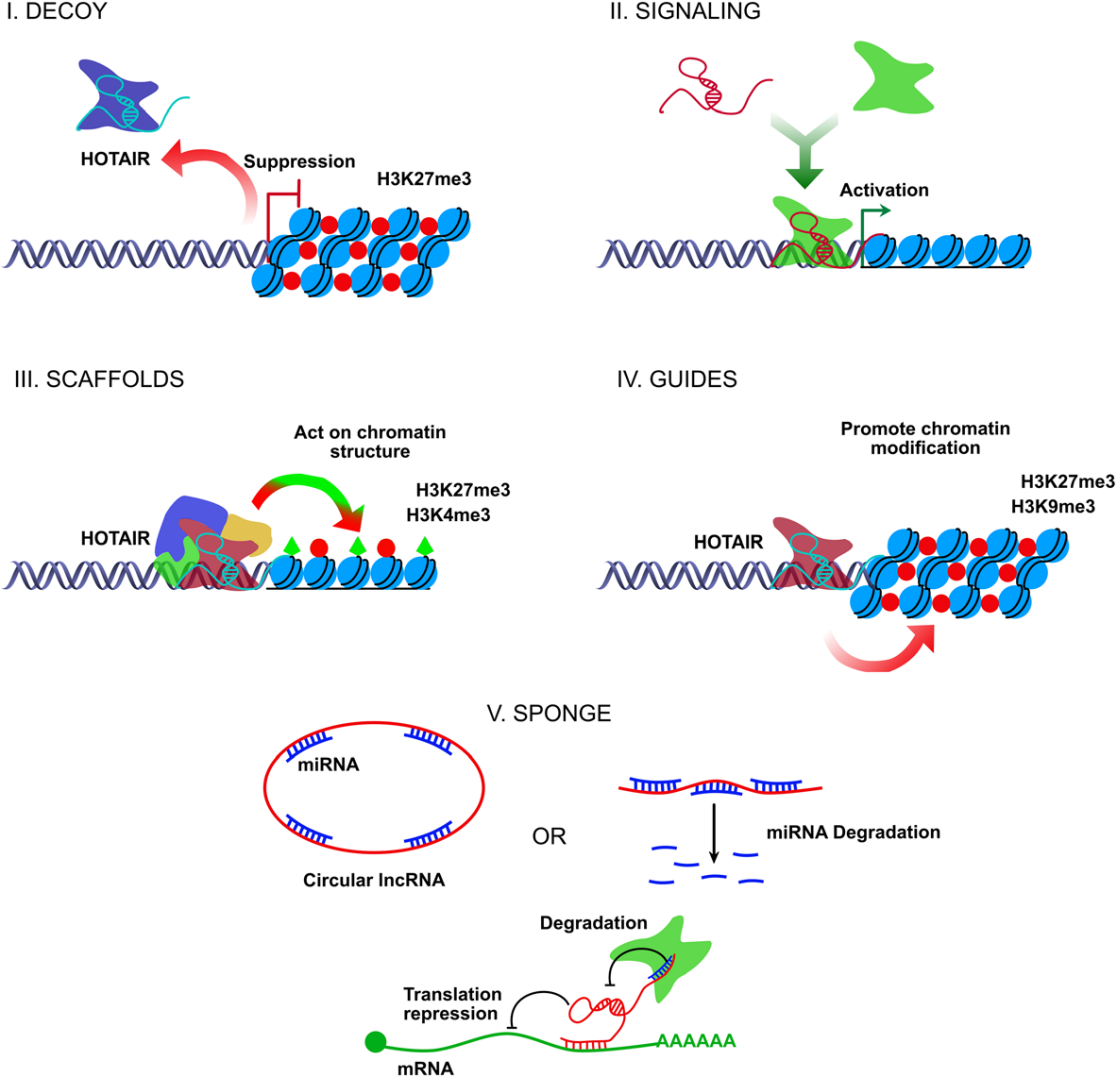
Five functional archetypes involved in the lncRNAs molecular mechanisms. **I.** Decoy: LncRNAs are transcribed and subsequently bound to the transcription factors, chromatin modifiers, or other regulatory factors outside from the chromatin structure, as well as moved in other nuclear subdomains preventing them from performing their biological effector function, free to perform any additional function, probably acting as a negative transcriptional regulator functionality. **II.** Signaling: LncRNAs are employed as molecular signals conducing to combinatorial actions of transcription factors and/or cell signaling pathways by regulating the space and time, on the stage of development and/or genetic expression patterns under certain cellular conditions by external stimuli. This archetype may act as biologically and functionally significant event markers at the cellular and/or tissue level of diseases events. **III.** Scaffolds: LncRNAs act as molecular platforms on which multiple proteins are assembled to form ribonucleoprotein complexes (RNPs). Each lncRNA-RNP complex may function in a structural manner by stabilizing complexes and controlling effector functions with an activating or repressive transcriptional activity or by altering post-translational modifications of the histone marks. **IV.** Guides: LncRNAs may recruit chromatin modifying proteins or remodeling enzymes to target specific genes, either in *Cis* position (near the site where lncRNA was transcribed or in neighboring regions) or in *Trans* position to target-distant genes into the chromatin specific sites. **V.** Sponge: LncRNAs that by complementarity of bases succeed in matching or sequestering sequences of small non-coding RNAs, such as miRNAs, are controlling the bioavailability of miRNAs versus lncRNAs themselves, with the functional biological repercussions at cellular level

Figure 2 represents a comprehensive summary of some of the lncRNAs archetypes functionality, which has been involved as genetic suppressors as lncRNAs Decoys, as well as lncRNAs activators, such as RNA signal, guide or scaffolding, playing an interacting role with a protein’s transcription factors profile or chromatin modifiers complexes. Additionally, they participate in the post-transcriptional regulation mechanisms of the mRNA maturation-edition-splicing process, and/or miRNAs decay process “Sponge”, and as a reservoir mechanisms for the small RNAs pool fraction [36].

Recent studies have identified large or small-scale mutation status profiles, as well as, several chromosomal translocations, copy number variations, nucleotides expansion, and/or single nucleotide polymorphisms (SNP), influencing the non-coding RNA transcription regions in the malignant tumor genome. In this regard, some reports have identified aberrant lncRNAs expression profiles associated or involved with different human malignant diseases [36–38]. In addition, in vitro experimental studies have described differential lncRNAs expression profiles between human histo-physiological normal cells and cancer cells from different histological epithelial origin, suggesting a significant role in the control of multiple molecular and cell signaling pathways, among others cell proliferation, migration and apoptosis [39].

Recently, some examples of the dysregulation of numerous lncRNAs have been reported such as PCAT-1 (Prostate cancer-associated transcript1), which is related with cell proliferation, migration, and invasion in NSCLC cells [40]; the overexpression of the lncRNA MALAT1 (Metastasis-Associated Lung Adenocarcinoma Transcript 1) associated with metastasis and poor prognosis [41, 42]; CARLo-5 (Cancer-Associated Region Long non-coding RNA) also associated with poor prognosis [43]; as well as the upregulation of H19, which promotes tumorigenesis in lung tissue [44], among others.

A typical example is the overexpressed lncRNA HOTAIR as a molecular factor of poor prognosis, based on several clinical reports. One clinical analysis based on a cohort of 42 lung cancer patients found a positive correlation with high invasion and metastasis ability, through in vitro and in vivo experimental strategies, respectively, using lung adenocarcinoma cells SPC-A1. Also, lncRNA HOTAIR has been reported playing a critical role in the physical interaction and recruitment of the Polycomb Repressor Complex-2 (PRC2) and LSD1/CoREST (REST co-repressor)/REST Complexes proteins; regarding these, some very exciting knowledge has been demonstrated relevant to the epigenetic regulation of one cytogenetic region at chromosome 12, promoting silencing of the genetic expression pattern of the HoxD cluster genes. This is based on the repressive histone mark H3K27me3, which occurs through the EZH2 enzymatic action and SUZ12 structural protein, as parts of the PRC2, as well as, by the histone demethylation process of the histone mark H3K4me2/3 through LSD1 enzyme, where lncRNA HOTAIR acts as a modular scaffold, in seeking higher ordered lncRNAs-epigenetic protein complex, and modifying histone profiles in human normal cells, but also in human malignant cells [45]. Some studies have confirmed how overexpressed lncRNAs guide chromatin remodeling complexes to specific genome positions, promoting modifying histone codes and/or chromatin remodeling activities, modulating genome-wide expression profiles in a temporary and/or in a permanent manner [36]. This is a summary of the chromatin remodeling complexes and lncRNAs "HOTAIR" functionality, which has previously been identified on some functional archetypes (Fig. 2), likely contributing to oncological chemoresistance mechanisms. Despite this, cancer-drug resistance mechanisms related to functional lncRNAs, as well as their pivotal role in cancer development, have not been fully elucidated. Recent evidence supports a fundamental role for lncRNAs, as molecular guides or scaffolds, as well as acting as a decoy or signal mechanism for the chromatin remodeler complexes at specific genome sites, controlling genetic expression by extensive and/or regional epigenetic changes through the human cancer genome [46, 47]. Previous reports have detected a significant percentage (38%) of the total well-known expressed lncRNAs in eukaryotic cells, attached to PRC2 complex and other chromatin repressor proteins complexes, such as CoREST and SMCX “Histone demethylase JARID1C” [48]. Although several lncRNAs have been identified to be joined to chromatin activators complexes as well, such as the Trithorax (TRX) protein complex and heterochromatin structures [49], they have additionally been described to be able to attach into several epigenetic enzymatic complexes as readers, writers, and/or erasers, functionally involved in the correct or aberrant PTMs code profiles [37, 50]. HOTTIP (HOXA transcript at the distal TIP), a lncRNA, has been identified to interact with WDR5 and/or WDR5/MLL protein complexes, which epigenetically controls the genetic transcription and expression of the HOXA cluster target genes, by a writing versus erasing mechanism of the histone activation mark H3K4me3. This previous information has been confirmed in human normal fibroblast cells [51]; however, as it occurs in malignant cells and chemo-resistant malignant cells, it is likely that lncRNAS are involved, as well.

### LncRNAs in malignant cells and cancer-drug resistance mechanisms

Oncological treatments commonly used on malignant diseases are based on cancer-drugs therapy protocols and/or target pharmacological agents that induce extensive DNA damage and/or blockade of the specific cell signaling pathways. However, utmost cancer-drugs currently used are not cancer-tissue- or genetic-specific, causing severe adverse effects on oncological patients including hematological toxicity, nephrotoxicity, nausea, and vomiting, among others [52].

Cisplatinum (CDDP; cis-diamminedichloro-platinum II) is one of the most potent antitumor agents, showing clinical activity against a wide variety of solid tumors [53]. Its cytotoxic mode of action induces the formation of adducts in DNA, which activates several cell signal transduction pathways, including those involving ATR, p53, p73, and MAPK, which culminates in the activation of apoptosis processes [53]. However, in spite of several cancer-drug based treatments, which do not have significant advantages over other pharmaco-oncological therapies [54], due to the ability of cancer cells to be resistant to cancer-drugs, such as CDDP, an impairment for a successful oncological therapy remains not resolved. To date, there is a continuing need to identify precise mechanisms involved and find new molecular targets to prevent cancer-drug resistance mechanisms [55, 56].

The emergence of cancer-drug resistance mechanisms represents a major oncological and biomedical issue, as it derives in the ineffectiveness of current therapies and treatment failure, as well as in the patient’s tumor relapse capacities [57]. Oncological resistance has been defined as malignant cell abilities to acquire, tolerate, and/or prevent chemotherapy effects resulting in the increased survival of the tumor cells populations, frequently leading to tumor recurrence after or during cancer-drug based therapies [58]. Chemoresistance of the malignant tumor cells to exposure of cancer-drug agents may occur in an innate manner, but tumor cells possess a unique genotype and have acquired several epigenetic characteristics providing a natural and/or an adapted ability to become resistant when exposed to one or multiple chemotherapeutic cancer-drugs [57, 59, 60].

Cisplatinum-based therapy protocols remain the most widely used chemotherapeutic agent as first- and/or second-line treatment protocols in lung cancer patients [61]. However, lung cancer patients have variable response rates to chemotherapy protocols affecting the efficacy of the cisplatinum cancer-drug, functionally affected by the acquired tumor cells chemoresistance mechanisms [62]. Tumor chemoresistance mechanisms remain not well characterized, but some genetic mutations located on coding-genes for repairing proteins could be responsible of chemoresistance phenomena, alterations on cellular apoptosis process through the ATR pathway, and/or p53-MAPK cell signaling pathways [63, 53], or by the functional activity of the membrane transporter proteins ABCG2/BCRP [64].

In fact, lncRNAs have previously been proposed as oncogenes or tumor suppressor genes functionally involved in tumorigenesis and cancer-drug resistance mechanisms. One of the lncRNAs previously involved in cisplatinum resistance has been HOTAIR, which is significantly overexpressed in cisplatinum-resistant A549/DDP cells. While HOTAIR-siRNA silencing assays have shown to partly re-sensitize A549/DDP cells to cisplatinum exposure in vitro and in vivo. In addition, it has been found that HOTAIR-mediated chemoresistance enhances cellular proliferation, inhibition of G0/G1 cell-cycle arrest, and apoptosis via p21^WAF1/CIP1^ (p21) expression regulation, which could mimic the effects of HOTAIR in the cisplatinum cancer-drug resistance within lung cancer cells. This confirms that HOTAIR upregulation contributes to the cisplatinum resistance of AD cells, at least in part, through the regulation of p21 gene expression [65].

In addition, studies by Fang and colleagues reported the overexpression of HOTAIR in Adriamycin-resistant SCLC cells (H69AR and H446 AR). Knock-down assays showed that HOTAIR inhibition increases the sensitivity of cell lines to doxorubicin, cisplatinum, and etoposide, increasing apoptosis and arrest of the cell cycle, suppressing tumor growth in vivo. In addition, an increase in DNA methylation of the Homeobox A1 (HOXA1) gene in chemo-resistant cells was correlated with increased expression levels of DNMT1 and DNMT3b, suggesting the role of HOTAIR in the chemoresistance of SCLC tumors by regulating the DNA methylation status in HOXA1 gene promoter [66].

On the other hand, Liu et al., reported the abnormal overexpression of HOTAIR in lung tumors of NSCLC patients resistant to cisplatinum-based treatment, as well as lung cancer resistant A549/DDP cells, confirming that HOTAIR is involved in cisplatinum resistance and involved in the expression induction of cancer stem-like cells biomarkers, such as β-catenin, Nanog, Oct3/4, SOX2, Klf4 (Krüppel-like factor 4), and c-Myc. Such molecules strongly associated with invasion, metastasis, poor prognosis, and cancer drug resistance [67] (See, Table 1).

**Table 1:** LncRNAs expression level and oncological treatment resistance in lung cancer

lncRNA	Possible targets	Mechanism	Drug	Dysregulation status	Lung cancer type	Reference
CUDR	Caspase 3	N/D	Doxorubicin/ Etoposide	Up	Squamous carcinoma	[68]
HOTAIR	p21	N/D	Cisplatinum	Up	NSCLC	[65]
	HOXA1	DNA methylation	Multidrug resistance (Cisplatinum, Adriamycin, Etoposide)	Up	SCLC	[66]
	Klf4	Promote stemness.	Cisplatinum	Up	NSCLC	[67]
AK126698	Wnt/β-catenin, NKD2	N/D	Cisplatinum	Down	NSCLC	[55]
MEG3	p53, Bcl-xl	N/D	Cisplatinum	Down	NSCLC	[62]
	Wnt/β-catenin	N/D	Cisplatinum	Down	NSCLC	[69]
NEAT1	CTR1	N/D	Cisplatinum	Down	Adenocarcinoma	[70]
H19	N/D	N/D	Cisplatinum	Up	NSCLC	[73]
ROR	PI3K/AKT/mTOR	N/D	Cisplatinum	Up	NSCLC	[71]
	EMT	N/D	Docetaxel	Up	NSCLC	[72]
CCAT1	let-7c	N/D	Docetaxel	Up	Adenocarcinoma	[75]
KCNQ1OT1	N/I	N/D	Paclitaxel	Up	NSCLC	[76]
TUG1	LIMK2b, EZH2	Promoter DNA methylation	Multidrug resistance (Cisplatinum, Adriamycin)	Up	SCLC	[74]

*Up* upregulated, *Down* downregulated, *SCLC* small-cell lung cancer, *NSCLC* non-small cell lung cancer, *N/D* non-determined

Another lncRNA named cancer upregulated drug-resistant (CUDR) has been involved in cancer-drug resistance through suppressing apoptosis. CUDR overexpression is induced in doxorubicin-resistant human squamous carcinoma cells which are resistant to cancer-drug-induced apoptosis. Overexpression of CUDR induces resistance to doxorubicin and etoposide, decreases drug-induced apoptosis, as well as expression and activity of caspase-3, promotes anchorage-independent growth in squamous carcinoma cells, demonstrating that CUDR may regulate cancer-drug sensitivity through caspase 3-dependent apoptosis [68] (See, Table 1).

Yang and colleagues in 2013 identified eight lncRNAs differentially expressed in A549/DDP cells. The downregulation of one of these lncRNAs, namely AK126698, in NSCLC cisplatinum-resistant cells suppressed the induction of apoptosis induced by cisplatinum in A549 cells, possibly through decreased naked cuticle homolog 2 (NKD2) expression and increased β-catenin expression, accumulation and nuclear translocation resulting in an altered WNT cellular signaling pathway and significantly depressed apoptosis induced by cisplatinum in lung cancer A549 cells. This previous information indicates that downregulation of this lncRNA AK126698 may contribute to chemo-resistance phenomena in NSCLC cells [55] (See, Table 1).

Liu and colleagues reported a MEG3 (Maternally Expressed 3 Gene) decreased expression in tumor tissues from patients insensitive to cisplatinum and in a cisplatinum-resistant A549 lung adenocarcinoma cells (A549/DDP), while the ectopic expression of MEG3 in A549/DDP may contribute to increased cisplatinum chemosensitivity through the regulation of p53 and Bcl-xl expression-induced mitochondria apoptosis pathway. So, the lncRNA MEG3 has been proposed as a tumor suppressor gene, able to induce a cancer drug cisplatinum-based sensitivity, as a DNA intercalating toxic mechanism in human lung cancer cells [62]. Xia et al., also reported a lower expression of MEG3 in cisplatinum-resistant A549 cells (A549/DDP) compared to parental A549 cells. Furthermore, MEG3 overexpression was able to re-sensitize A549/DDP cells to cisplatinum in vitro, and the downregulation of MEG3 enhanced sensitivity to cisplatinum in lung cancer cells through activation of the Wnt/β-catenin signaling pathway. This increase could be associated with an arrest in the cell cycle and increased apoptosis [69] (See, Table 1), suggesting that MEG3 may have an important role in the acquired cisplatinum-resistance in NSCLC.

Jian and colleagues on 2016 reported that lncRNA NEAT1 (Nuclear Enriched Abundant Transcript 1) is involved in the positive control expression of copper transporter CTR1 (copper uptake protein 1), which has previously been reported in atypical function and subcellular location, promoting chemo-resistance in cancer cells, whereas CTR1 overexpression may increase cisplatinum-sensitivity [70] (See, Table 1).

Recently, Shi and colleagues in 2017 reported that inhibited expression by shRNAs of the lncRNA ROR (regulator of reprogramming) in chemo-resistant lung cancer cells A549/DDP correlates with decreased expression of mRNA and protein of the PI3K/AKT/mTOR signaling pathway and Bcl-2, while it relates to increased expression of the pro-apoptotic Bax protein, which promotes sensitivity to cisplatinum-induced apoptosis, inhibits cellular proliferation, as well as migration and invasion in vitro and in vivo. This confirms that lncRNA ROR promotes resistance to cisplatinum in lung AD histological type through PI3K/AKT/mTOR signaling pathway [71] (See, Table 1).

Additionally, Pan et al., have reported that the increased lncRNA ROR expression is also associated with docetaxel chemoresistance in lung AD cells (SPC-A1/DTX and H1299/DTX), while a decrease in the ROR expression re-sensitized chemo-resistant lung AD cells to docetaxel - treatment, decreasing cell proliferation, migration, and invasion in vitro and in vivo, additionally increasing apoptosis, as well as expression of the epithelial biomarkers E-cadherin and β-catenin, also contributing to the expression of lncRNA ROR in the epithelial mesenchymal transition (EMT) process in lung tumor cells (See, Table 1). Moreover, miRNA 145 (miR-145) has been identified as a direct target of the lncRNA ROR [72], representing an example of the functional sponge archetype carried out by lncRNAs (Fig. 2).

On the other hand, in 2017, Wang et al., have reported overexpression of lncRNA H19 in lung cancer cells A549/DDP, while knockdown assays allowed the restoration of the sensitivity of A549/DDP cells to cisplatinum, increasing apoptosis, and arrest of the cell cycle (G0/G1), while decreasing cellular migration. In this case, the increase in the lncRNA H19 expression correlated with poor disease-free progression rates, while being associated to clinical progression in lung cancer patients with higher malignancy in accordance to TNM classification, leading to higher metastasis indexes in correlation with negative response to cisplatinum-based therapy [73] (See, Table 1).

Another such example is lncRNA TUG1 (Taurine upregulated gene 1), whose overexpression in SCLC chemo-resistant cells (H69AR, H446DDP), as well as in lung solid tumors derived from SCLC patients, correlates with advanced clinical stages and poor survival. However, KD assays in chemo-resistant SCLC cells reduce cell proliferation and increase sensitivity in vivo for Adriamycin, cisplatinum, and Etoposide-based treatments. In addition, transcriptome analysis identified the overexpression of LIMK2b (LIM Kinase 2B), in a positive correlation with lncRNA TUG1, in both chemo-resistant lung cancer cells and solid lung SCLC tumors, whereas such expression was regulated through EZH2 catalytic activity, reducing levels of the histone mark H3K27me3 through the LIMK2b gene promoter sequences [74] (See, Table 1).

Scientific information is growing spectacularly regarding unknown archetype functionality of the majority of lncRNAs, associated or not to treatment resistance. However, several evidence continues showing data on new lncRNAs, such as CCAT1 (Colon Cancer-Associated Transcript-1) overexpressed in lung AD tumors, and docetaxel resistant cells (SPC-A1/DTX and H1299/DTX), promoting both cellular proliferation and apoptosis resistance mechanisms, as well as induction of the EMT process using in vitro and in vivo assays [75] (See, Table 1).

Additionally, miRNAs such as let-7c have shown to be negatively correlated with lncRNAs such as CCAT1, suggesting a functional sponge archetype between miRNAs and lncRNAs, as shown in Fig. 2, regulating, as a whole, sensitivity or resistance to cancer-drug docetaxel in NSCLC tumors [75], while lung AD patients and AD A549/PA cells resistant to paclitaxel have shown overexpression of the lncRNA KCNQ1OT1 (KCNQ1 Opposite Strand/Antisense Transcript 1) in correlation with tumor growth, poor histological differentiation, positive lymphatic metastasis, and advanced TNM clinical stage. Meanwhile cancer-drug resistance ability has been shown to be dependent on the multidrug resistance receptor 1 (MDR1), but regulated by lncRNA CCAT1 [76] (See, Table 1).

According to the above mentioned, it is likely that several lncRNAs are epigenetically controlling common molecular pathways, among others DNA repairing mechanisms, cell cycle, and cellular apoptosis through ATR and p53-MAPK cell signaling pathways, and cellular cycle arrest mechanisms. However, specific gene target therapies involved in efficient lung oncological therapies have not been systematically analyzed, probably suggesting a need for novel future directions focusing on well-known but new efficient pharmacological therapy protocols and/or gene target therapies, such as those based on EGFR cellular signaling pathways in lung cancer research.

However, possible molecular mechanisms involved in chemo-resistance capacities remain elusive. Therefore, additional research should be undertaken in order to identify the mechanisms involved and to provide better therapy strategies for the cancer-drugs and gene-target therapies "EGFR" clinically used in the treatment of NSCLC patients.

### LncRNAs and EGFR-tyrosine kinase inhibitors (EGFR-TKIs)-treatment resistance

Recent studies have identified and proposed several lncRNAs likely involved as novel gene target therapy resistance mechanisms. In lung cancer, as well in other malignant carcinomas diseases, gene target-based therapies have emphasized the use of tyrosine kinase inhibitors (TKIs), focusing on a priority oncological gene target, the epidermal growth factor receptor (EGFR). TKIs are used worldwide, they include, among others, erlotinib, gefitinib, afatinib, etc., some of which have been reported to improve both clinical outcome and quality-of-life in lung cancer patients.

However, oncological therapy efficacy has been limited by the innate-primary and/or adaptive-acquired therapy resistance mechanisms, which usually emerge after the first year of the applied oncological therapeutic treatment [77, 78].

In a classical scenario the mechanisms involved in acquired resistance to TKIs include a second-site mutation in the threonine residue 790 by a methionine (T790 M), seen in approximately 60% of total cases, c-MET gene amplification (5–10%), PIK3CA mutations (~ 5%), and mutations in the BRAF gene (~ 1%) associated with small-cell lung carcinoma transformation process derived from adenocarcinoma tumors and/or epithelial-origin tumors involved in a mesenchyme transition (EMT) process with ~ 5% of total cases. However, to date, the TKIs resistance mechanisms involved, reported in approximately 30% of the total lung cancer cases remain unknown [78, 79].

**Fig. 3:**
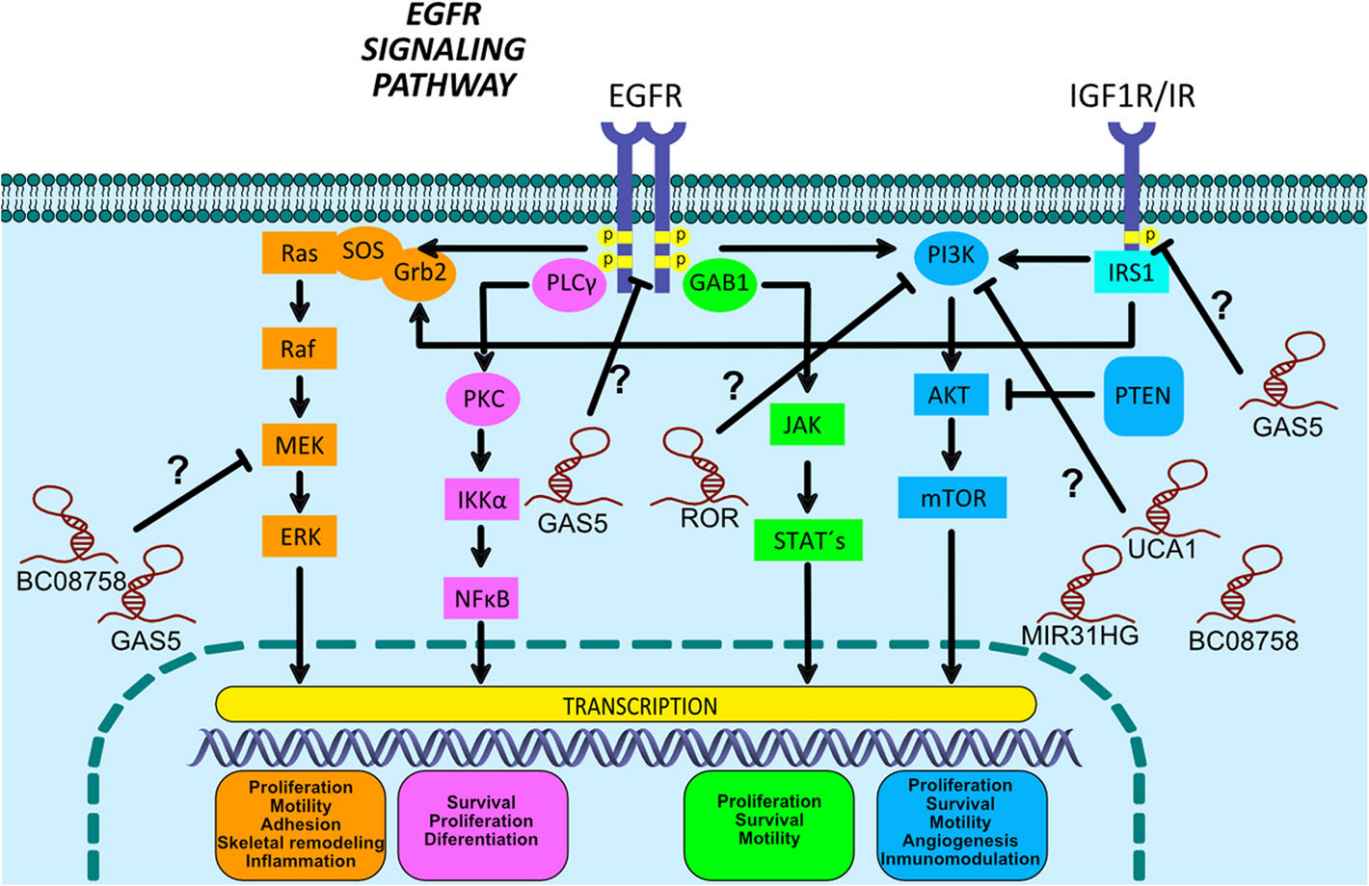
EGFR cell signaling and lncRNAs relationship in lung cancer therapy resistance. The binding of the EGF ligand to the EGFR receptor causes the autophosphorylation of the tyrosine residues located in the terminal COOH-domain, allowing the activation of multiple downstream signaling cascades through the recruitment of signaling proteins to the intracellular portion of the receptor including Ras/Raf/MEK/ERK, PI3K/Akt, JAK/STAT and PLC, which ultimately drive to proliferation, survival, and/or tumor cell invasion. Growth factor-stimulated "IGF-1R" also induces the activation of AKT and ERK signaling pathways. Likewise, the probable participation of lncRNAs along the EGFR cell signaling pathway has been indicated, as a new likely lncRNAs therapy targets in lung cancer

In addition to the above-described mechanisms, Cheng and colleagues on 2015 reported UCA1 (Urothelial Carcinoma Associated 1) overexpression in lung cancer cells with acquired resistance to gefitinib (PC9/R and H1975), as well as in 37 lung cancer patients with acquired resistance to EGFR-TKIs therapy with exon 19 deletion and/or genetic mutation in exon 21 (L858R) status. These abnormalities were detected after the TKIs based-treatment was applied. In these patients, UCA1 overexpression was associated with a shorter progression-free survival in lung cancer patients without T790M genetic mutation status. Moreover, using in vivo KD-assays, it was identified that decreased UCA1 expression partially sensitizes lung cancer cells to gefitinib-based therapy, increasing apoptosis rate, and also showed a smaller tumor size compared with the negative control treatment group in resistant gefitinib lung cancer cells (PC9/R). Besides, in silico analysis, using Kyoto databases (KEGG) have identified an enrichment of the mTOR cell signaling pathway (Fig. 3), suggesting its involvement in EGFR-TKIs resistance mechanisms. An additional protein analysis showed a positive correlation between decreased UCA1 expression with the epithelial marker E-Cadherin over-expression and attenuated expression of mesenchymal gene markers N-cadherin, Snail, and Vimentin [78]. The research conducted by Cheng and colleagues identified the role of UCA1 overexpression in cancer cells or small TKIs resistant cells [78]. Further KD assays confirmed that lncRNA UCA1 promotes resistance to gefitinib through the functioning of the AKT/mTOR cellular signaling pathway, suggested in Fig. 3 [78] (See Table 2).

**Table 2:** LncRNAs expression level and TKIs treatment resistance in lung cancer

lncRNA	Possible targets	Mechanism	Drug	Dysregulation status	Lung cancer type	Reference
UCA1	AKT/mTOR	N/D	TKIs	Up	NSCLC	[78]
	N/I	N/D	Gefitinib	Up	NSCLC	[85]
GAS5	IGR-1R	N/D	Gefitinib	Down	NSCLC	[81]
MIR31HG	EGFR/PI3K/AKT signaling pathway	N/D	Gefitinib	Up	NSCLC	[84]
BC087858	PI3K/AKT, MEK/ERK	N/D	EGFR-TKIs	Up	NSCLC	[82]
	N/I	N/D	Gefitinib	Up	NSCLC	[80]
BC200	N/I	N/D	Gefitinib	Up	NSCLC	[80]
HOTAIR	N/I	N/D	Gefitinib	Down	NSCLC	[80]
MALAT1	N/I	N/D	Gefitinib	Down	NSCLC	[80]
ENST00000507437	N/I	N/D	Gefitinib	Up	NSCLC	[80]
ENST00000508827	N/I	N/D	Gefitinib	Up	NSCLC	[80]
NR_026685	N/I	N/D	Gefitinib	Up	NSCLC	[80]
ENST00000381279	N/I	N/D	Gefitinib	Down	NSCLC	[80]
ENST00000418077	N/I	N/D	Gefitinib	Down	NSCLC	[80]
BG188549	N/I	N/D	Gefitinib	Down	NSCLC	[80]
BE244504	N/I	N/D	Gefitinib	Down	NSCLC	[80]
CASC9	N/I	N/D	Gefitinib	Up	NSCLC	[85]
EWAST1 (LINC00277)	N/I	N/D	Gefitinib	Down	NSCLC	[85]
LINC00524	N/I	N/D	Gefitinib	Down	NSCLC	[85]

*Up* upregulated, *Down* downregulated, *SCLC* small-cell lung cancer, *NSCLC* non-small cell lung cancer, *N/I* non-identified, *N/D* non-determined

Additionally, Cheng and colleagues reported a large number of differentially expressed lncRNAs (1731 upregulated, while 2936 downregulated) in gefitinib-sensitive and gefitinib-resistant PC9 lung cancer cells. While bioinformatic analyses have revealed that several lncRNAs aberrantly expressed probably play important roles in regulating EGFR-TKIs resistance through several cellular proliferation and apoptosis mechanisms. In this regard, overexpressed lncRNAs H19 and BC200 have previously been validated; in contrast, HOTAIR and MALAT1 have been found to be downregulated in gefitinib-resistant lung cancer cells, suggesting that such differential expression patterns for several lncRNAs are frequently involved in EGFR-TKIs treatment resistance mechanisms in human NSCLCs [80] (See Table 2).

In a different sense, lncRNAs can also be involved in the inability of some cells to develop TKI-resistance. A study from 2015 reported that a decreased lncRNA GAS5 expression was associated and/or involved in the lack of resistance capacity when lung cancer was exposed to gefitinib-based therapy [81]. The study identified a decreased GAS5 expression in lung AD tissues and in lung AD cells A549, and H1299 EGFR-wild-type, as well as EGFR-mutated H1975 (T790 M) and HCC827 (E746-A750 deletion) lung cancer cell lines. Moreover, overexpressed GAS5 combined with gefitinib-treatment therapy reduced tumor growth in vitro and in vivo and confirmed that IGF-1R (Insulin-Like Growth Factor 1 Receptor) is a downstream key mediator, with a negative correlation with GAS5 expression, and involved with the physiological phosphorylated levels of the p-EGFR, p-Akt, and p-ERK proteins (Fig. 3). Such proteins are known to be functionally implicated in cellular signaling activation pathways in human lung tumors [81] and likely playing a role in the clinical evolution of lung cancer (See Table 2).

In addition, another lncRNA called BC087858 has been associated to TKI resistance mechanisms, identified in gefitinib-resistant PC9/R and NSCLC cell lines (H1975, PC9/R, PC9/G2), as well as in 78 advanced primary lung carcinomas with EGFR exon 19 deletion (19DEL) or exon 21-point mutation (L858R) status. Overexpression of the lncRNA BC087858 correlates with lower survival rate, under TKIs-based therapy, while siRNAs genetic silencing assays in gefitinib-resistant lung cancer cells promoted gefitinib-sensibility, associated to E-cadherin gene overexpression, and low gene expression for Vimentin, ZEB1, and Snail, as well as a decreased level of phosphorylated EGFR-, AKT-, and ERK. This suggests that overexpressed lncRNA BC087858 might be functionally involved in the TKI-gefitinib acquired resistance mechanisms through a dysfunctional activation of the PI3K/AKT, MEK/ERK cell signaling pathways, and EMT process in lung cancer [82]. However, in spite of the growing body of evidence, the mechanisms likely involved are not yet fully characterized (Fig. 3) (See Table 2).

Besides, Wu et al. have generated an EGFR-TKI-resistant HCC827-8-1 cell line, analyzing gene expression patterns compared to its parental HCC827 cell line, identifying a total of 1476 deregulated lncRNAs in the EGFR-TKI-resistant cell line. Functional analysis indicated that several lncRNAs may be involved in cellular pathways associated to EGFR-TKIs, including focal adhesion, cell cycle, cellular proliferation, and apoptosis, playing important roles in EGFR-TKIs resistance mechanisms through *cis*- and/or *trans*-regulation of target protein-coding genes [83] (See Table 2).

However, studies by Wang and colleagues identified the differential expression of lncRNAs such as PVT1, H19, MIR31HG, BOK-AS1, CBR3-AS1, and LincRNA-p21, being MIR31HG significantly expressed in gefitinib-resistant NSCLC cells (PC9 versus PC9-R). However, transfected PC9-R cells by si-MIR31HG developed a greater sensitivity to gefitinib, probably mediated by the inhibition of EGFR/PI3K/AKT pathway and p53 activation. They also induced cellular apoptosis through the mitochondrial apoptosis pathway, which leads to cell cycle arrest at G2/M phase, while overexpression of lncRNA MIR31HG contributes to the gefitinib resistance in PC9-R cells, affecting cellular proliferation, apoptosis, and cell cycle control, through the EGFR/PI3K/AKT cellular pathway, mentioned in Fig. 3 and included in Table 2 [84].

In another study Ma et al., analyzed the expression profile of lncRNAs in NSCLC cell lines (PC9, HCC827 and H3122), resistant to EGFR-TKIs treatment, in contrast to parental cell lines, and using the Gene Expression Omnibus (GEO) analyses, identified thousands of deregulated lncRNAs, highlighting the overexpressed lncRNA CASC9, whereas the lncRNA EWAST1 (LINC00227) showed its involvement in the sensitivity to gefitinib. In addition, GEO analyses determined the enrichment of important cellular signaling pathways, including cell proliferation, apoptosis, and chromatin assembly [85] (See Table 2).

In addition to all the above-mentioned information, previous reports have focused on a limited vision, exploring lncRNAs expression profiles and potential biological function but in most cases with an unknown archetype. However, in an effort to overcome such limitation, Xue and colleagues have analyzed transcriptional signatures related to cancer-drug resistance and constructed a functional dual co-expression network based on lncRNA-mRNAs profiles and levels. They have, therefore, identified 101 lncRNAs co-expressed with 324 mRNAs, differentially expressed between multi-resistant and parental lung cancer cell lines. A detailed bio-informatics network analysis showed a co-expression sub-network related to oncological cancer-drug resistance mechanisms, associated to the clinical efficacy and/or poor survival in lung cancer patients [86] (See Table 2).

### Perspectives

#### LncRNAs-mRNAs co-expression, chromatin remodeling complexes, and histone code aberrations involved in lung oncological therapy resistance

All the information previously described along with several other findings have strongly confirmed how some lncRNAs profiles can predict either sensitivity and/or resistance capacity to cisplatinum-based and/or TKI-based treatment therapies. As such, lncRNAs are proposed as key molecular markers for individualized oncological therapeutics, allowing us to improve the response in lung cancer therapy protocols. Besides, in lung cancer, additional mechanisms have recently been associated to EGFR-TKIs resistance capacity, such as those focusing on the Sonic Hedgehog (SH) cell signaling pathway, which has recently been implicated in cancer stem cell-like functionality, although the molecular mechanisms involved have not been completely identified. In this sense, Chellappan’s research group on 2015, determined SH components, such as GLI1, involved in the SOX2 protein control expression through a GLI1 physical interaction with SOX2 gene promoter sequences, suggesting a key transcriptional role involved in treatment resistance and poor clinical prognosis in NSCLC patients. Moreover, inhibition or removal of some SH-GLI1 pathway components seems to cooperatively work together with the EGFR-cell signaling resistance in TKIs-based protocols reducing lung tumor cellular viability [87].

Finally, a probable connection with EGFR-TKIs therapy efficacy may be associated as it has recently been demonstrated between lncRNA SOX2-OT overexpression in lung SCC versus lung AD histological types, associated with a poor survival rate in lung cancer patients (studying a cohort of 83 lung cancer patients) and involved in lung cancer cell proliferation and SOX2 coding gene overexpression. It is important to highlight that the SOX2 gene is genetically located adjacent to the lncRNA SOX2-OT genetic sequences [88]. A positive co-expression between lncRNA SOX2-OT and mRNA SOX2 expression has previously been demonstrated in breast cancer cell lines and breast tumor derived from patients, identified by a large study of 1106 breast cancer patients [89]. In addition, it was recently identified that lncRNA GLI1AS expression level is negatively co-expressed with the mRNA GLI-1 in rhabdomyosarcoma, pancreatic carcinoma, medulloblastoma, prostatic carcinoma, gastric carcinoma, and lung carcinomas, indirectly controlling tumor growth and tumor cell proliferation seen in xenotransplant in vivo assays. This suggests a probable positive transcriptional biofeedback between GLI-1 mRNA and protein on lncRNA GLI1AS expression on epithelial origin tumors [90], the latter probably epigenetically involved in cancer therapy resistance, including lung cancer.

In addition, it has very recently been established how lncRNA HOTAIR is functionally involved in lung tumor progression through PRC2, and how is epigenetically associated to cancer-drug cisplatinum resistance mechanisms [65, 91]. However, additional studies must be conducted to establish the precise key role of lncRNAs in targeted therapies, such as EGFR-TKIs, associated to functional epigenetic mechanisms among others, PRC2 (EZH2 and SUZ12 proteins) and/or TRX Activator Complex (SWI/SNF/CoREST proteins), directly involved to the PTMs of the histone code in cancer epigenomics.

Based on the above-mentioned, preliminary evidence suggest that a co-expressed profile, for instance lncRNA SOX2-OT and coding gene SOX2 [88], as well as, lncRNA GLI1AS and coding gene GLI1 [90], are probably not only involved in EGFR-TKIs-based therapy resistance mechanisms in lung cancer, but also in epigenetic mechanisms involved in cancer-drug (cisplatinum)-based therapy resistance. This, as we have recently identified, induced epigenetic changes on GLI-1 gene promoter sequences, based on the enriched bivalent histone marks H3K4me3 and H3K9me3, as well as activated RNA Pol II promoted in a cisplatinum dependent-manner [92], as probably occur for lncRNAs promoter sequences in others, as SOX2-OT. This is a likely explanation to lung tumor cell proliferation and invasion rates, where an increased or diminished SOX2-OT expression level appears to be involved in the recruitment of TRX versus PRC2 complexes, as has previously been reported to occur with the lncRNA HOTAIR (Fig. 2), through the recruitment of EZH2 enzymatic activity for the establishment of the histone repressive mark H3K27me3 or H3K4me2/3 by TRX complexes [45], promoting epigenetic silencing at p21 gene sequences promoter [91] and causing a decreased expression of tumor suppressor protein p21 involved in cellular proliferation and cell cycle control [65]. Aside from that, it has previously been reported how SOX2-OT overexpression correlates with malignant stem-cell like phenotype, positively associated with the SOX2 and POU5F1 (also known as OCT4) protein level expression, poor overall survival, and poor therapy efficacy in lung cancer patients [88], likely in correlation with a transient recruitment balance among TRX versus PRC2. However, lncRNAs functionality in gene target therapies have not been totally described, where additional basic and clinical studies are required in the future lung clinical oncology and lung cancer biomedicine epigenetics research, as the leading cause of cancer deaths worldwide.

## Conclusion

In conclusion, histone PTMs accompanied by a lncRNAs expression profile and function have been epigenetically associated and/or involved in several mechanisms, as a scaffold for the recruitment of writers, erasers, and/or readers of PTMs and involved in chromatin remodeling processes controlling genome expression. They have also been involved in epigenetic adaptation processes of the oncology therapy resistance. In turn, this information may be employed in order to propose molecular predictors of high therapeutic sensibility, which will allow biomedical and oncology experts to make better clinical decisions into identifying patients with a higher resistance capacity when receiving cancer-drug cisplatinum-based and/or target gene therapies. This screening process would prove to be beneficial for lung cancer patients. A likely lncRNAs profile will include, among others, HOTAIR, MEG3, NEAT1, UCA1, and/or AK126698 in cancer-drugs cisplatinum-based therapies, and the recently proposed GAS5, UCA1, BC087858 and SOX2-OT involved in the gene-target therapies as EGFR-TKIs based oncological treatments (Fig. 3). However, the epigenetic mechanisms remain to be elucidated in human lung cancer therapies.

## Abbreviations

AD: Adenocarcinomas
EGFR: Epidermal growth factor receptor
KD: Knock-down
lncRNAs: Long non-coding RNAs
NSCLC: Non-small cell lung carcinomas
PRC2: Polycomb Repressive Complex-2
PTMs: Post-Traslational Modifications
SCC: Squamous Cell Lung Carcinomas
TKIs: Tyrosine Kinase Inhibitors
TRX: Trithorax Activator Complex
